# Socioeconomic Inequalities in Childhood Undernutrition in India: Analyzing Trends between 1992 and 2005

**DOI:** 10.1371/journal.pone.0011392

**Published:** 2010-06-30

**Authors:** Malavika A. Subramanyam, Ichiro Kawachi, Lisa F. Berkman, S. V. Subramanian

**Affiliations:** 1 Center for Integrative Approaches to Health Disparities, School of Public Health, University of Michigan, Ann Arbor, Michigan, United States of America; 2 Department of Society, Human Development and Health, Harvard School of Public Health, Boston, Massachusetts, United States of America; 3 Center for Population and Development Studies, Harvard School of Public Health, Boston, Massachusetts, United States of America; UCL Institute of Child Health, United Kingdom

## Abstract

**Background:**

India experienced a rapid economic boom between 1991 and 2007. However, this economic growth has not translated into improved nutritional status among young Indian children. Additionally, no study has assessed the trends in social disparities in childhood undernutrition in the Indian context. We examined the trends in social disparities in underweight and stunting among Indian children aged less than three years using nationally representative data.

**Methods:**

We analyzed data from the three cross-sectional rounds of National Family Health Survey of India from 1992, 1998 and 2005. The social factors of interest were: household wealth, maternal education, caste, and urban residence. Using multilevel modeling to account for the nested structure and clustering of data, we fit multivariable logistic regression models to quantify the association between the social factors and the binary outcome variables. The final models additionally included age, gender, birth order of child, religion, and age of mother. We analyzed the trend by testing for interaction of the social factor and survey year in a dataset pooled from all three surveys.

**Results:**

While the overall prevalence rates of undernutrition among Indian children less than three decreased over the 1992–2005 period, social disparities in undernutrition over these 14 years either widened or stayed the same. The absolute rates of undernutrition decreased for everyone regardless of their social status. The disparities by household wealth were greater than the disparities by maternal education. There were no disparities in undernutrition by caste, gender or rural residence.

**Conclusions:**

There was a steady decrease in the rates of stunting in the 1992–2005 period, while the decline in underweight was greater between 1992 and 1998 than between 1998 and 2005. Social disparities in childhood undernutrition in India either widened or stayed the same during a time of major economic growth. While the advantages of economic growth might be reaching everyone, children from better-off households, with better educated mothers appear to have benefited to a greater extent than less privileged children. The high rates of undernutrition (even among the socially advantaged groups) and the persistent social disparities need to be addressed in an urgent and comprehensive manner.

## Introduction

India is experiencing a rapid economic boom due in part to the opening of its markets in the 1990s and the emergence of a knowledge-based economy [1]. However, this prosperity has not translated into well-being among the country's young children. The prevalence of underweight (a widely used indicator of undernutrition) among children under age five in India is one of the highest in the world—43% in 2006—surpassed only by Bangladesh, Yemen and Timor-Leste [2]. India is home to 55 million of the world's underweight children under age five—about one-third of the global burden of underweight in this age group [3]. During the prosperous 1990s, the average rate of decline in prevalence of underweight has been around 0.9% per year among Indian children aged below five years [3] whereas in China, another Asian country with a rapidly growing economy, it declined by approximately 5% per year [4].

The nutritional status of young children is an important indicator of health and development—it is not only a reflection of past health insults but an important indicator of future health trajectories [5]. Children under age three are particularly vulnerable to undernutrition, and because the growth rate in this period is greater than any other age period, it increases the risk of growth retardation [6]. Furthermore, undernutrition among young children captures the extent of development in a society [5] and is thus a marker for the overall well being of a population. It is well established that socioeconomic factors such as lower levels of household wealth and maternal education are important causes of childhood undernutrition [5].

Few studies have analyzed patterns of distribution of childhood undernutrition in India, across social factors such as household wealth and maternal education, using nationally representative data [7]–[9]. However, these were all cross sectional analyses and we did not find any published study that has evaluated the pattern of social disparities in childhood undernutrition in India across time. Studying the trend in social inequalities in childhood undernutrition over a period of economic growth sheds light on the potential benefits and adverse effects of such growth on this vulnerable population, thereby allowing us to review economic policies and their implementation from a public health perspective.

We therefore examined trends in social disparities in undernutrition among Indian children under age three over the period of 1992—2005, using national-level data on child nutritional status from three cross-sectional surveys conducted in 1992, 1998 and 2005.

## Methods

### Ethics statement

The data were analyzed anonymously, using publicly available secondary data, therefore no ethics statement is required for this work.

### Data

**Figure 1:**
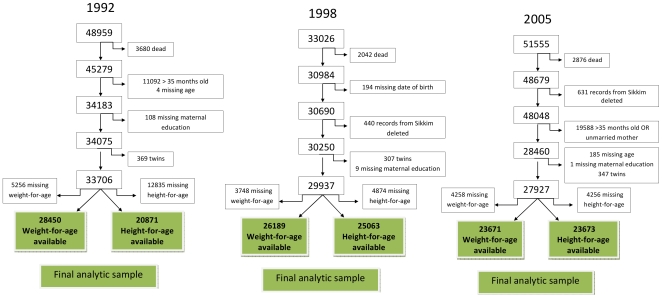
The scheme of arriving at the final analytic sample size using data from the National Family Health Surveys of India 1992, 1998 and 2005.

We analyzed data from the three cross-sectional rounds of National Family Health Survey (NFHS) of India held in 1992, 1998 and 2005 to examine the trends in social disparities in undernutrition among children less than three years of age. These surveys used a multi-stage cluster sampling design to collect data on fertility, mortality, family planning, and important aspects of nutrition, health, and health care. The NFHS is the Indian version of the Demographic and Health Surveys (DHS) which use standard model questionnaires designed for, and widely used in, developing countries [10]. Details of these nationally representative surveys have been described elsewhere [11]. We used data from the interviews with women of reproductive age which includes information about their children. As the 1992 and 1998 surveys included only those children who were under age three and born to those women in the household who were interviewed, the 2005 data were restricted to children meeting these two criteria. Data on Sikkim were missing in 1992 and data from Tripura were missing in 1998, therefore we excluded these states from our analytic sample. The original sample size from the three surveys, inclusion and exclusion criteria, and the resulting analytic sample size for each survey are shown in Figure 1. The individual response rate for women was 96.1% in 1992, 95.5% in 1998 and 94.5% in 2005.

Following convention, we operationalized undernutrition as four binary variables denoting (a) underweight, (b) severely underweight, (c) stunted and (d) severely stunted. We used the World Health organization (WHO) standards to compute the Z scores for weight-for-age and height-for-age [12]. Following WHO guidelines, children with a Z score of less than −2 on these variables were classified as underweight and stunted, respectively. Children whose Z scores were less than −3 were classified as severely underweight and severely stunted. Typically, weight-for-height and its binary equivalent wasting are also used to report the nutritional status of a population. However, wasting is highly influenced by seasonality, and not appropriate for use as an indicator to compare undernutrition rates in a population over time [13]. We therefore did not include wasting in our analysis. The height and weight of the children were measured by one researcher on each survey team. Height was measured to the nearest 0.1 cm using a measuring board, taking care to measure length for children less than 2 years of age. Weight was measured using the UNICEF Uniscale to the nearest 100 grams.

### Predictor Variables

The predictors of interest in this study were: household wealth, maternal education, caste, and urban residence. We chose these variables because social epidemiological theories [5], as well as previous studies [7]–[9] indicate that they are important determinants of undernutrition among young children in India.

Household wealth was operationalized as possession of household assets e.g., television, mattress, cattle. The dataset contained an index of household assets that had been created by ORC Macro using principal components analysis (PCA) on items related to possession of assets [14]. This method was developed by Filmer and Pritchett and the index has been found to correlate highly with income data in developing countries [15]. We weighted the PCA scores in the dataset by the household sampling weights to ensure that the distribution was representative of all the households in India and then divided the households into quintiles. Since we were interested in the relative disparity among different wealth quintiles in each year, the quintiles were created separately for each survey.

We constructed the maternal education variable using data on the number of years of schooling with the following categories: no education (0 years of schooling), primary education (1–5 years), secondary education (6–12 years) and greater than secondary (>12 years of education).

We operationalized the caste variable as having the following categories: Scheduled caste, Scheduled tribe, “Other” caste and “missing/no” caste. This classification (using terminology adopted by the Government of India) focuses more on the socially disadvantaged castes, and all privileged caste groups are represented in the “Others” group [16]. The census of India definition of urban/rural [17] was used to classify a household as urban or not.

### Covariates

The association between undernutrition and the predictor variables of interest was assessed after accounting for the following covariates: age in months, gender, birth order of child, religion, and age of mother (all as reported by the mother.)

### Analysis Plan

Prevalence of underweight, severe underweight, stunted, and severely stunted were calculated for each survey period using survey analysis methods that account for sampling design. In order to account for the multilevel structure of the data (children nested within households within clusters nested in states) and to account for clustering, we used a logistic multilevel modeling approach, with random effects specified for households, clusters and states. Specifically, we modeled the log odds of undernutrition for child *i* in household *j*, cluster *k*, nested in state *l*.

In order to measure the trend in social disparities over time, we assessed whether the association between predictor of interest and undernutrition varied by survey year. This assessment required us to pool the data from all three years and test for interactions between the predictor of interest and the survey year in the pooled dataset using Wald tests. We additionally fit our final models stratified by survey year. The final models for all four outcomes included household wealth, maternal education, caste, and urban residence (predictors of interest), as well as age in months, gender, birth order of child, religion, and age of mother. For presentation, we report the estimates and standard errors as model-based predicted probabilities (PP), along with their 95% confidence intervals (CI.) We also present disparities in undernutrition using the prevalence ratio (PR) [18], which is a better estimate than the odds ratio when the outcome is not a rare event. All analyses were conducted using SAS 9.1(SAS Institute, Cary, NC, USA) and MLwiN 2.10 [19].

## Results

The overall prevalence (%) of underweight was 49.14, 43.82 and 40.26 in 1992, 1998 and 2005 respectively. The corresponding prevalence (%) of stunting was 52.43, 50.65, and 44.73. The bivariate distributions of the prevalence of underweight, severe underweight, stunting and severe stunting are given in Table 1.

**Table 1:** Bivariate distribution of the prevalence of underweight, severe underweight, stunting and severe stunting among children aged less than three in the final analytic sample.

		Frequency (%)			Frequency (%)		
Characteristic	Category	Total for underweight	Underweight	Severely underweight	Total for stunting	Stunted	Severely stunted
Survey year	1992–93	28450	12821 (49.14)	5854 (23.27)	20871	10360 (52.43)	5827 (30.52)
	1998–99	26189	10617 (43.82)	4652 (19.57)	25063	12219 (50.65)	6785 (28.13)
	2005–06	23671	8235 (40.26)	3033 (15.57)	23671	9628 (44.73)	4575 (21.91)
Age (in months)	0 to 11	26159	8539 (36.06)	3594 (15.27)	23124	6295 (28.53)	2898 (12.73)
	12 to 23	26638	11457 (47.70)	4933 (21.20)	23666	12507 (55.95)	6745 (30.57)
	24 to 35	25513	11677 (50.06)	5012 (22.37)	22815	13405 (62.66)	7544 (36.41)
Gender	Male	40445	16856 (45.57)	7168 (19.98)	35898	17054 (49.89)	9285 (27.42)
	Female	37865	14817 (43.49)	6371 (19.18)	33707	15153 (47.96)	7902 (25.52)
Birth order	First	23385	8073 (39.23)	3257 (15.85)	20878	8544 (43.98)	4173 (21.84)
	Second	20887	7870 (40.79)	2944 (16.73)	18678	8130 (46.31)	4121 (23.60)
	Third	13579	5622 (45.45)	2269 (19.29)	11985	5799 (50.35)	3100 (27.04)
	Fourth	8169	3746 (50.75)	1691 (23.15)	7238	3711 (53.43)	2105 (30.95)
	Fifth and greater	12290	6362 (55.02)	3378 (28.81)	10826	6023 (57.74)	3688 (36.08)
Maternal age	13 to 16 years	796	406 (52.68)	191 (23.12)	571	359 (53.21)	184 (26.92)
	17 to 19 years	6509	2935 (46.72)	1326 (20.65)	4834	2670 (48.33)	1406 (25.40)
	20 to 24 years	29328	11661 (42.66)	4840 (17.99)	22579	11954 (47.97)	6168 (24.75)
	25 to 29 years	24670	9490 (43.04)	3887 (18.32)	19220	9755 (47.43)	5268 (26.14)
	30 and more	17007	7181 (49.01)	3295 (23.98)	13401	7469 (53.35)	4161 (31.16)
Maternal education	No schooling	38252	19519 (53.40)	9374 (26.00)	33036	18352 (56.82)	10969 (33.97)
	Primary	12296	5002 (43.82)	1852 (16.66)	10707	5131 (48.97)	2580 (24.61)
	Secondary	22354	6290 (31.48)	2057 (10.46)	20802	7533 (38.35)	3201 (16.24)
	>Secondary	5408	862 (18.50)	256 (5.78)	5070	1191 (24.47)	437 (8.93)
Household wealth	Richest quintile	14814	3154 (23.01)	989 (7.42)	13672	4059 (30.35)	1610 (11.62)
	Second quintile	17491	5828 (35.29)	2044 (12.62)	15990	6722 (43.14)	3172 (20.33)
	Third quintile	16242	6894 (45.03)	2826 (18.49)	14266	7047 (49.92)	3821 (26.90)
	Fourth quintile	14999	7489 (52.08)	3429 (24.09)	13081	7072 (54.89)	4069 (31.07)
	Poorest quintile	14764	8308 (57.34)	4251 (29.09)	12596	7307 (58.96)	4515 (36.21)
Caste	Scheduled caste	12717	5972 (49.22)	2646 (22.12)	11523	6086 (54.24)	3435 (30.82)
	Scheduled tribe	11023	4505 (54.28)	2143 (27.49)	9788	4582 (53.69)	2570 (30.89)
	No caste	1194	373 (38.84)	121 (13.80)	1189	454 (44.61)	202 (20.53)
	Other caste	53376	20823 (42.25)	8629 (18.04)	47105	21085 (47.09)	10980 (24.95)
Type of residence	Urban	23588	7485 (34.92)	2779 (13.48)	21553	8433 (41.03)	4009 (19.61)
	Rural	54722	24188 (47.51)	10760 (21.47)	48052	23774 (51.39)	13178 (28.61)
Religion	Hindu	57549	24594 (45.29)	10549 (20.01)	50451	23937 (49.30)	12877 (26.79)
	Muslim	11357	4652 (44.82)	2043 (19.92)	10163	4868 (50.54)	2664 (27.99)
	Christian	5889	1358 (28.09)	480 (9.17)	5631	2033 (35.64)	1012 (18.18)
	Sikh	1886	538 (29.15)	200 (10.23)	1840	694 (37.51)	301 (16.16)
	Other	1629	531 (43.00)	267 (19.33)	1520	675 (48.82)	333 (22.36)
Total		78310	31673 (44.56)	13539 (19.59)	69605	32207 (48.96)	17187 (26.50)

### Results of stratified multivariable multilevel analyses

**Figure 2:**
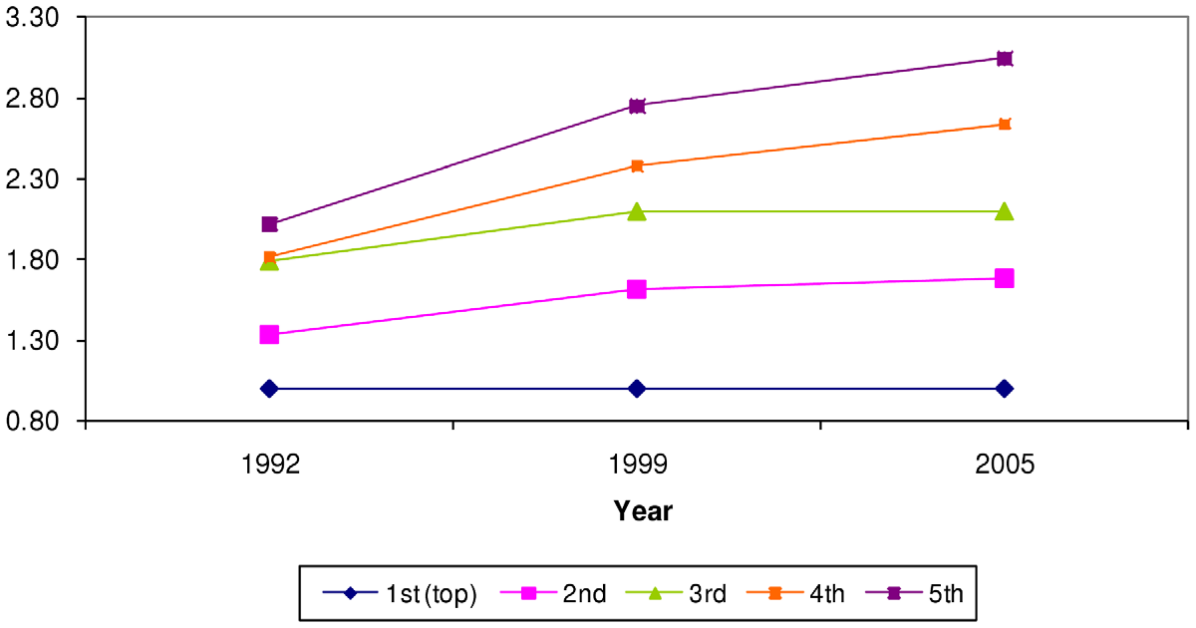
Prevalence ratio (PR) of being severely underweight by quintiles of household wealth. X axis = survey year. Y axis = prevalence ratio. Blue rhomboid = richest quintile, reference group (PR = 1). Pink square = second quintile. Green triangle = third quintile. Orange square = fourth quintile. Purple square = poorest quintile. PR from model adjusting for age in months, gender, birth order of child, age of mother, religion, household wealth, maternal education, caste, and urban residence. P value for Wald test of interaction between survey year and wealth quintiles (8 degrees of freedom) = 0.015.

Household wealth, caste, and maternal education were significant predictors of all four undernutrition outcomes. There was no evidence to suggest that urban residence was associated with undernutrition once household wealth, maternal education and caste were included in the model. Predicted probabilities of being underweight, severely underweight, stunted or severely stunted are shown in Tables 2 and 3. Figures 2–5 display the trends in the disparities in severe underweight and severe stunting across categories of wealth and maternal education. These results are from models accounting for household wealth, maternal education, caste, and urban residence (predictors of interest), as well as age in months, gender, birth order of child, religion, and age of mother. All tests of interactions refer to interactions of the particular social factor with the survey year.

**Figure 3 pone-0011392-g003:**
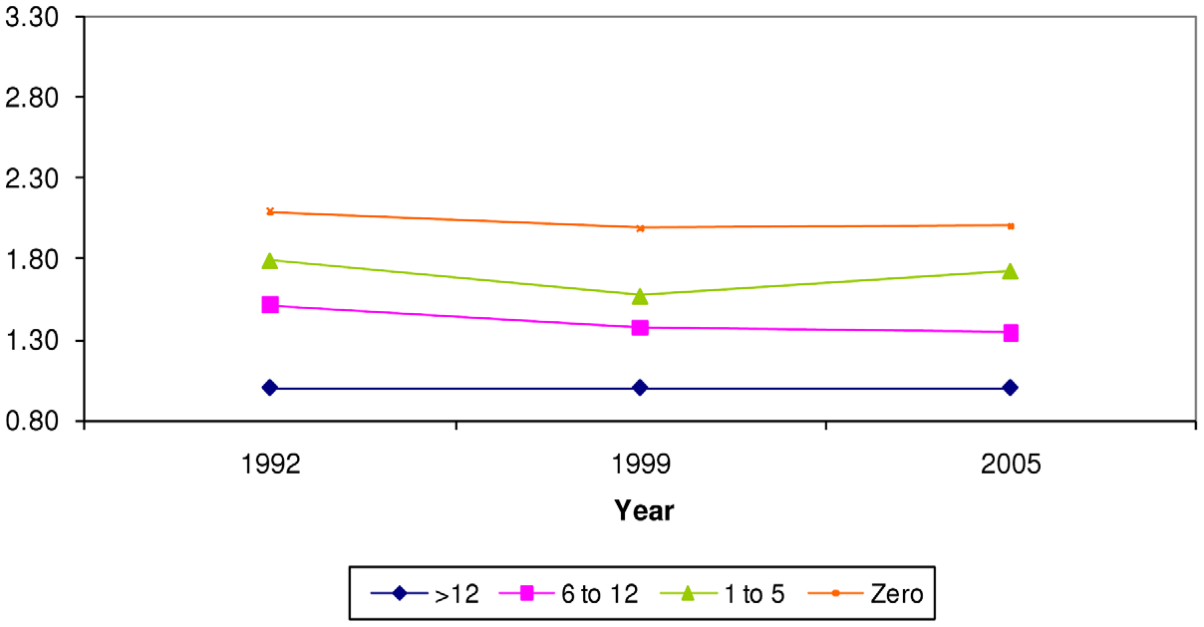
Prevalence ratio (PR) of being severely underweight by maternal education. X axis = survey year. Y axis = prevalence ratio. Blue rhomboid = >secondary education (>12 years), reference group (PR = 1). Pink square = secondary education (6 to 12 years). Green triangle = primary education (1 to 5 years). Orange square = no schooling (zero years). PR from model adjusting for age in months, gender, birth order of child, age of mother, religion, household wealth, maternal education, caste, and urban residence. P value for Wald test of interaction between survey year and maternal education (6 degrees of freedom) was >0.05.

**Figure 4 pone-0011392-g004:**
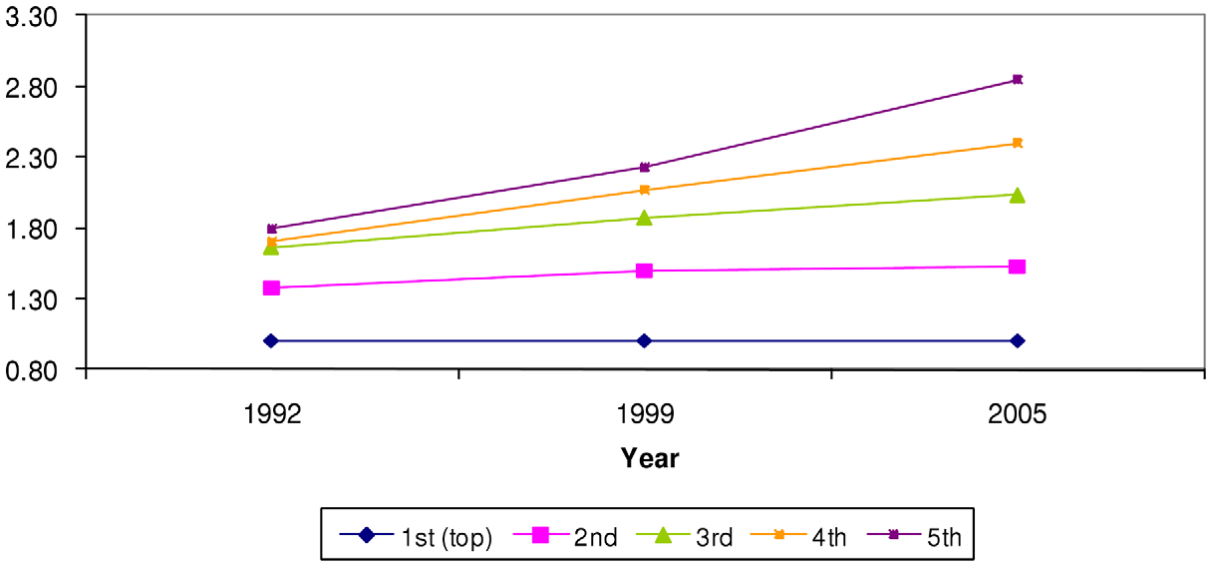
Prevalence ratio (PR) of being severely stunted by quintiles of household wealth. X axis = survey year. Y axis = prevalence ratio. Blue rhomboid = richest quintile, reference group (PR = 1). Pink square = second quintile. Green triangle = third quintile. Orange square = fourth quintile. Purple square = poorest quintile. PR from model adjusting for age in months, gender, birth order of child, age of mother, religion, household wealth, maternal education, caste, and urban residence. P value for Wald test of interaction between survey year and wealth quintiles (8 degrees of freedom) = 0.053.

**Figure 5:**
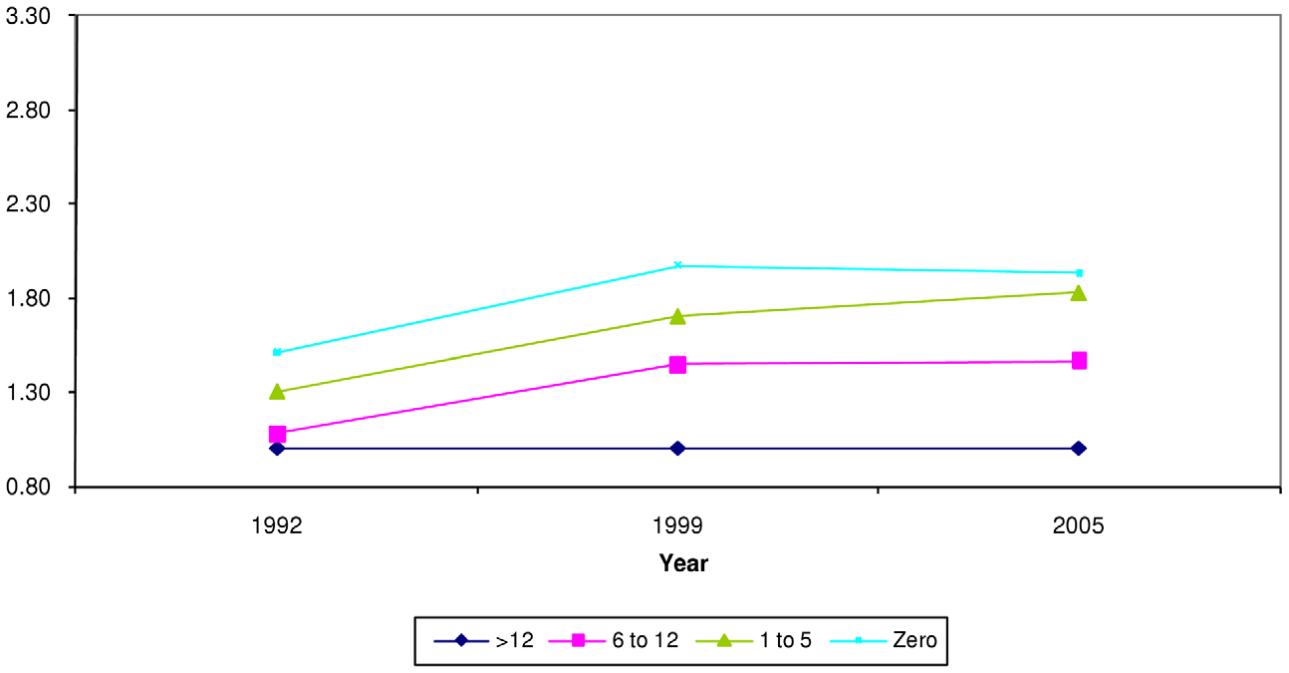
Prevalence ratio (PR) of being severely stunted maternal education. X axis = survey year. Y axis = prevalence ratio. Blue rhomboid = >secondary education (>12 years), reference group (PR = 1). Pink square = secondary education (6 to 12 years). Green triangle = primary education (1 to 5 years). Orange square = no schooling (zero years). PR from model adjusting for age in months, gender, birth order of child, age of mother, religion, household wealth, maternal education, caste, and urban residence. P value for Wald test of interaction between survey year and maternal education (6 degrees of freedom) = 0.022.

**Table 2:** Predicted probabilities (95% confidence intervals) for underweight and severely underweight across categories of social determinants over the three survey years (N = 78310).

		Underweight			Severely underweight		
Variable	Subcategory	1992	1998	2005	1992	1998	2005
Gender	Male	0.45 (0.40, 0.50)	0.40 (0.35, 0.45)	0.34 (0.30, 0.38)	0.17 (0.14, 0.21)	0.12 (0.09, 0.15)	0.12 (0.09, 0.14)
	Female	0.40 (0.35, 0.45)	0.38 (0.33, 0.42)	0.32 (0.28, 0.36)	0.15 (0.12, 0.18)	0.12 (0.09, 0.14)	0.11 (0.09, 0.14)
Household wealth	Richest quintile	0.29 (0.25, 0.34)	0.24 (0.21, 0.28)	0.19 (0.17, 0.23)	0.10 (0.08, 0.13)	0.06 (0.04, 0.07)	0.06 (0.04, 0.07)
	Second quintile	0.38 (0.33, 0.43)	0.34 (0.30, 0.38)	0.28 (0.25, 0.32)	0.13 (0.11, 0.17)	0.09 (0.07, 0.11)	0.09 (0.07, 0.12)
	Third quintile	0.45 (0.40, 0.50)	0.40 (0.35, 0.45)	0.34 (0.30, 0.38)	0.18 (0.14, 0.22)	0.12 (0.09, 0.15)	0.12 (0.09, 0.14)
	Fourth quintile	0.46 (0.41, 0.52)	0.43 (0.38, 0.48)	0.40 (0.36, 0.45)	0.18 (0.14, 0.22)	0.13 (0.10, 0.17)	0.15 (0.12, 0.18)
	Poorest quintile	0.48 (0.43, 0.54)	0.47 (0.42, 0.52)	0.43 (0.38, 0.48)	0.20 (0.16, 0.25)	0.15 (0.12, 0.19)	0.17 (0.13, 0.21)
Caste	Scheduled caste	0.50 (0.45, 0.56)	0.39 (0.35, 0.44)	0.39 (0.35, 0.44)	0.20 (0.16, 0.25)	0.13 (0.11, 0.17)	0.14 (0.11, 0.17)
	Scheduled tribe	0.47 (0.41, 0.53)	0.38 (0.33, 0.43)	0.38 (0.33, 0.43)	0.19 (0.15, 0.24)	0.14 (0.11, 0.18)	0.15 (0.12, 0.19)
	No caste	NA*	0.37 (0.31, 0.42)	0.37 (0.31, 0.42)	NA*	0.14 (0.09, 0.21)	0.10 (0.07, 0.14)
	Other caste	0.45 (0.40, 0.50)	0.34 (0.30, 0.38)	0.34 (0.30, 0.38)	0.17 (0.14, 0.21)	0.12 (0.09, 0.15)	0.12 (0.09, 0.14)
Maternal education	No schooling	0.47 (0.42, 0.52)	0.43 (0.38, 0.47)	0.36 (0.31, 0.40)	0.20 (0.16, 0.24)	0.15 (0.12, 0.18)	0.13 (0.11, 0.17)
	Primary	0.45 (0.40, 0.50)	0.40 (0.35, 0.45)	0.34 (0.30, 0.38)	0.17 (0.14, 0.21)	0.12 (0.09, 0.15)	0.12 (0.09, 0.14)
	Secondary	0.37 (0.33, 0.43)	0.34 (0.30, 0.38)	0.30 (0.26, 0.33)	0.14 (0.11, 0.18)	0.10 (0.08, 0.13)	0.09 (0.07, 0.11)
	>Secondary	0.27 (0.22, 0.34)	0.27 (0.22, 0.31)	0.22 (0.18, 0.26)	0.10 (0.06, 0.14)	0.07 (0.05, 0.10)	0.07 (0.05, 0.09)
Type of residence	Urban	0.45 (0.40, 0.50)	0.40 (0.35, 0.45)	0.34 (0.30, 0.38)	0.17 (0.14, 0.21)	0.12 (0.09, 0.15)	0.12 (0.09, 0.14)
	Rural	0.43 (0.38, 0.48)	0.38 (0.34, 0.42)	0.33 (0.30, 0.37)	0.16 (0.13, 0.19)	0.12 (0.09, 0.14)	0.11 (0.09, 0.13)

*The category of “no caste” was not available in 1992.

**Table 3:** Predicted probabilities (95% confidence intervals) for stunted and severely stunted across categories of social determinants over the three survey years (N = 69605).

		Stunted			Severely stunted		
Variable	Subcategory	1992	1998	2005	1992	1998	2005
Gender	Male	0.56 (0.52, 0.61)	0.50 (0.45, 0.55)	0.42 (0.38, 0.46)	0.29 (0.24, 0.33)	0.25 (0.21, 0.29)	0.20 (0.17, 0.23)
	Female	0.52 (0.47, 0.56)	0.48 (0.43, 0.53)	0.39 (0.35, 0.43)	0.25 (0.21, 0.30)	0.23 (0.19, 0.27)	0.17 (0.14, 0.20)
Wealth quintile	Richest quintile	0.42 (0.38, 0.47)	0.34 (0.30, 0.39)	0.26 (0.23, 0.29)	0.17 (0.14, 0.21)	0.13 (0.11, 0.16)	0.10 (0.08, 0.12)
	Second quintile	0.51 (0.46, 0.55)	0.44 (0.39, 0.49)	0.35 (0.32, 0.39)	0.24 (0.20, 0.28)	0.20 (0.17, 0.23)	0.15 (0.13, 0.18)
	Third quintile	0.56 (0.52, 0.61)	0.50 (0.45, 0.55)	0.42 (0.38, 0.46)	0.29 (0.24, 0.33)	0.25 (0.21, 0.29)	0.20 (0.17, 0.23)
	Fourth quintile	0.57 (0.53, 0.62)	0.54 (0.49, 0.59)	0.54 (0.49, 0.58)	0.29 (0.25, 0.34)	0.27 (0.23, 0.32)	0.24 (0.20, 0.28)
	Poorest quintile	0.59 (0.54, 0.63)	0.55 (0.50, 0.60)	0.50 (0.45, 0.55)	0.31 (0.26, 0.36)	0.29 (0.25, 0.35)	0.28 (0.24, 0.33)
Caste	Scheduled caste	0.59 (0.54, 0.64)	0.56 (0.51, 0.61)	0.46 (0.42, 0.51)	0.32 (0.27, 0.37)	0.29 (0.24, 0.34)	0.24 (0.20, 0.27)
	Scheduled tribe	0.56 (0.50, 0.61)	0.54 (0.48, 0.59)	0.43 (0.38, 0.47)	0.28 (0.23, 0.33)	0.28 (0.23, 0.33)	0.22 (0.18, 0.26)
	No caste	NA*	0.50 (0.40, 0.60)	0.43 (0.37, 0.48)	NA*	0.25 (0.21, 0.29)	0.20 (0.17, 0.23)
	Other caste	0.56 (0.52, 0.61)	0.50 (0.45, 0.55)	0.42 (0.38, 0.46)	0.29 (0.24, 0.33)	0.25 (0.21, 0.29)	0.19 (0.15, 0.24)
Maternal education	No schooling	0.58 (0.53, 0.62)	0.53 (0.48, 0.57)	0.43 (0.39, 0.48)	0.33 (0.29, 0.38)	0.29 (0.25, 0.33)	0.21 (0.18, 0.25)
	Primary	0.56 (0.52, 0.61)	0.50 (0.45, 0.55)	0.42 (0.38, 0.46)	0.29 (0.24, 0.33)	0.25 (0.21, 0.29)	0.20 (0.17, 0.23)
	Secondary	0.47 (0.43, 0.52)	0.45 (0.40, 0.50)	0.38 (0.34, 0.41)	0.24 (0.20, 0.28)	0.21 (0.18, 0.25)	0.16 (0.14, 0.19)
	>Secondary	0.40 (0.35, 0.47)	0.37 (0.32, 0.42)	0.29 (0.25, 0.33)	0.22 (0.17, 0.28)	0.15 (0.12, 0.18)	0.11 (0.08, 0.14)
Type of residence	Urban	0.56 (0.52, 0.61)	0.50 (0.45, 0.55)	0.42 (0.38, 0.46)	0.29 (0.24, 0.33)	0.25 (0.21, 0.29)	0.20 (0.17, 0.23)
	Rural	0.55 (0.51, 0.59)	0.51 (0.46, 0.55)	0.40 (0.36, 0.44)	0.27 (0.23, 0.31)	0.24 (0.20, 0.28)	0.18 (0.15, 0.21)

*The category of “no caste” was not available in 1992.

### Trends in disparities by wealth

There was a clear gradient in the association between wealth quintiles and the probability of being undernourished, greater household wealth was associated with lower probabilities of all four outcomes. This pattern was seen for all survey years. Figure 2 displays the prevalence ratio of severe underweight across wealth quintiles over time (p for interaction = 0.015.) Figure 4 displays prevalence ratios for severe stunting (p for interaction = 0.053.) As can be seen from Table 2, while the probability of being severely underweight has decreased for *all* wealth groups between 1992 and 1998, this decrease was of smaller magnitude between 1998 and 2005. However, Figure 2 shows that wealth-based disparities on the ratio scale increased over time. The ratio of predicted probability in the poorest quintile to that in the richest quintile was 2 in 1992, 2.5 in 1998 and 2.8 in 2005. A similar pattern was seen for severely stunted (Figure 4), underweight (not shown) and stunting (not shown).

### Trends in disparities by maternal education

Just as observed with wealth quintiles, there was a clear gradient in the association between maternal education and the probability of being undernourished. Children of mothers with greater education had lower likelihood of being undernourished. Figure 3 displays the trends in the prevalence ratio of being severely underweight across categories of maternal education over the three survey years (p for interaction >0.05.) The probability of being severely underweight decreased for children of *all* maternal education groups between 1992 and 1998; however the rates appear static between 1998 and 2005 (Table 2). The disparities on a ratio scale did not change much across the survey years. For severe stunting, however, the pattern was different—there was a decrease in predicted probability (Table 3) and a slight widening of disparities over time (Figure 5), with the prevalence ratios of 1.5 in 1992, 1.9 in 1998 and 1.9 in 2005 (p for interaction = 0.022.)

### Trends in disparities by caste

Children belonging to Scheduled Castes and Scheduled Tribes were statistically more likely to be undernourished as compared to children from the socially privileged Other caste in each of the three periods, however the effect size was small. The general trend was of decreasing probability of being severely underweight and severely stunted for *all* caste groups over the three periods. There was no significant trend in the disparities on a ratio scale by caste.

## Discussion

This examination of social disparities in undernutrition among Indian children aged less than three years using nationally representative survey data from 1992, 1998 and 2005 presented a complex picture. We discuss three major findings. First, the overall prevalence rates of undernutrition in India decreased over the 1992–2005 period; second, social disparities in undernutrition over these years either widened or stayed the same, however the absolute rates of undernutrition have decreased for everyone regardless of their social status; and third, the disparities by household wealth were greater than the disparities by maternal education.

### Trends in undernutrition

This study found that there was a steady decrease in the rates of stunting in the 1992–2005 period, while the decline in underweight was greater between 1992 and 1998 than between 1998 and 2005. Numerous factors might have influenced this decline in stunting including, for example, the rapid economic growth India has experienced between 1990 and 2007, the provision of primary health care at the national level resulting in improved health of girl children over generations (leading to better long-term nutritional status of their offspring) and implementation of preventive nutrition programs such as the Integrated Child Development Services Scheme [2]. Although the declining trend is a positive finding, it needs to be tempered by the fact that the absolute rates of undernutrition in India continue to be higher than the majority of developing countries [2]. One study compared the trends in stunting among underfives in China and India during the 1990s and found that the rate of stunting in India remained almost unchanged while it halved between 1992 and 1998 in China [4]. A simple calculation using UNICEF data for 1994 and 2000–2006 allows us to compare the decline in undernutrition among underfives among various countries (see Appendix S1). India appears to lag behind comparable countries in reducing underweight, but has experienced some success, relative to others, in decreasing stunting over the years.

### Trends in social disparities in undernutrition

We found that social disparities in undernutrition either widened or were static, but never narrowed, against a background of national economic growth. Disparities in underweight and stunting by household wealth were progressively wider when we compared surveys over the 1992–2005 period. Even as we saw a lowering of undernutrition among all wealth groups, children from better-off families experienced a greater decline in undernutrition between 1992 and 2005 compared to children from households in the lowest wealth quintile, and these disparities widened over time. When viewed in a context of decreasing poverty but increasing income inequality [20], these findings suggest that children from better-off families have benefited from the economic growth to a greater extent than children from poor families. Rising income inequality has been linked to underinvestment in human capital because it leads to lack of attention and resources towards programs that benefit the poor and a greater focus on policies that benefit the well-off, and this adversely affects the health status of the poor [21].

Disparities in undernutrition by levels of maternal education were generally similar to wealth based disparities. Although slightly widened for stunting, disparities have stayed the same for underweight. The period of 1992–2005 has seen a rapid spread in the reach of mass media as well as a broadening in the content of media messages [22] and given the reach of satellite television [23] and mobile telephones in India [24], we might expect greater health dividends at every level of education. Notably, disparities in stunting, a marker of chronic undernutrition, appeared to widen over time. However, the rates of stunting have decreased at *all* levels of maternal education. We can conclude that the advantages of economic growth might be reaching children irrespective of their mother's education, although children of better-educated mothers appear to have benefited to a greater extent.

There was no change in the pattern of the caste based disparities in undernutrition between 1992 and 2005. Additionally, the association between caste and undernutrition in each year, while statistically significant, was of extremely small magnitude once household wealth and maternal education were accounted for. This implies that inequalities by wealth and education account for most of the caste-based disparities in undernutrition. While Bharati et al [9] report that children from historically disadvantaged caste groups had, on average, lower levels of weight-for-age and height-for-age, our findings are not directly comparable to their study because they pool all disadvantaged caste groups together while we look at them as separate categories.

Remarkably, we did not find any evidence for differential rates of undernutrition based on urban residence or gender after accounting for household wealth, maternal education, caste and other factors. These findings correspond with the results reported by Bharati et al [9], even though their operationalization of nutritional status differs from ours.

A review of literature revealed only one study that examined *trends* in social disparities in child undernutrition in a developing country setting. This study used nationally representative data from Cameroon from 1991 and 1998 [25] and found that household economic status and maternal education were positively associated with weight-for-age and height-for-age *Z* scores among children less than 3 years of age. The study also found that, between 1991 and 1998, the beneficial effect of maternal education strengthened and the gap between the richest and the poorest groups increased, although these results did not attain statistical significance. Our results correspond with those of the Cameroon study, though they used Z scores to assess nutritional status while we used binary variables.

### Household wealth versus maternal education

A comparison of the trend in inequalities over time shows us that disparities by household wealth (Figures 2 and 4) were greater than those by maternal education (Figures 3 and 5.) The trend was of increasing wealth based inequalities in both underweight and stunting whereas maternal education based disparities widened slightly for stunting. Additionally, the differentials on the ratio scale as well as the absolute difference in probabilities show that increasing wealth-based disparities are of greater magnitude than maternal education based disparities. This suggests that a reduction in economic disparities might lead to a great reduction in social disparities in undernutrition. This comparison underscores the need to critically examine the manner in which economic growth at the national level is affecting the economic status of various groups in the population.

### Limitations

A few limitations need to borne in mind while interpreting the results of this study. First, our interpretations frequently refer to the economic growth in India as a proximal determinant of social disparities in undernutrition, though our study is not designed to examine the effects of economic growth on these disparities. However, to our knowledge, this is the first study to describe the trends in *social disparities* in undernutrition in this population in the background of rapid economic growth. Second, we acknowledge the large amount of missing data on undernutrition outcomes in our study. However, a comparison of the sociodemographic distribution among those missing and not missing data showed that our analytic sample is very similar to those missing data. We therefore believe that our findings are not affected to a great extent by the missingness; however, this remains an issue and needs to borne in mind while interpreting the results. Given that this is the only nationally representative source of data on undernutrition among young children, and is used by national and international agencies [2], we are using the best data that are available. Therefore, we think that the results of this study are generalizable to all children under age three across India. Another limitation is that we were unable to explore differences, if any, between the Other Backward Classes (OBC) caste group and the privileged “Other” caste group. Since the 1992 data contained only three caste groupings- SC, ST and Other, we were forced to pool OBC and the Other group in the 1998 and 2005 data. Similarly, data limitations prevented us from using a finer classification of the urban residence variable, such as large cities, smaller towns and villages. Fourth, education was measured as years of schooling, and might not be comparable over time. For example, a high school education in 1992 might be very different from a high school education in 2005. However such differences, if any, would have resulted in a non-differential bias. Also, we were able to compare how children from wealthier families differed from less wealthy families but unable to study the relationship between absolute levels of wealth and undernutrition. While trends in disparities by absolute levels of wealth are important, the NFHS is not the best source of data for such an analysis. However, we believe that the trends presented in this study raise important questions about the underlying causes for the widening wealth based disparities in undernutrition during a period of rapid economic growth.

### Conclusion

This study displayed trends in social disparities in childhood undernutrition in India using data from a time when India began experiencing major economic growth. We would expect this growth to have increased household income, decreased food insecurity and improved the quality of nutrients available to Indians. Supporting this argument, we see that rates of undernutrition decrease over the 14 years, across all social groups. However, this decrease was unequal across categories of household wealth and maternal education. Notably, no narrowing of social disparities was observed in this study, despite using underweight and stunting, two indicators that capture both acute and chronic effects of undernutrition. The high rates of undernutrition (even among the socially advantaged groups) and the persistent social disparities need to be addressed in an urgent and comprehensive manner.

## Supporting Information

Appendix S1 (0.04 MB DOC)
