# Supplementary material for: Socioeconomic Inequalities in Childhood Undernutrition in India: Analyzing Trends between 1992 and 2005
Source: PLoS One. 2010 Jun 30;5(6):e11392. doi: 10.1371/journal.pone.0011392 (PMC2894973; doi:10.1371/journal.pone.0011392)
Supplement: Appendix S1 — (0.04 MB DOC) [file pone.0011392.s001.doc]

**Appendix S1**

Data on rates of underweight (moderate + severe) and stunting (moderate + severe) for the various countries were obtained from the UNICEF reports: State of the World’s Children, 1994 and State of the World’s Children 2006. The difference in the rate was divided by a denominator of 10 since the exact year of data collection was not known for the figures after 2000. Countries which liberalized trade around the same time as India did and had underweight and stunting rates similar to that of India were selected for this comparison. China was added to this list because we compare India and China in the introduction.

| Country | Underweight | | | Stunting | | |
| --- | --- | --- | --- | --- | --- | --- |
|  | 1994 | 2000-2006 | Decline  (% per year) | 1994 | 2000-2006 | Decline  (% per year) |
| India | 53.4 | 48 | 0.54 | 53 | 43 | 1.00 |
| Indonesia | 40 | 28 | 1.20 |  |  |  |
| Pakistan | 40 | 38 | 0.20 | 50 | 37 | 1.30 |
| Nigeria | 36 | 29 | 0.70 | 43 | 38 | 0.50 |
| Philippines | 34 | 28 | 0.60 |  |  |  |
| China | 17 | 7 | 1.00 | 32 | 11 | 2.10 |

We see that in 1994, the prevalence (%) of underweight among underfives was 53.4, 40, 40, 36 and 34 in India, Indonesia, Pakistan, Nigeria and Philippines, respectively. The decline in underweight in this period was 0.54% per year for India compared to 1.2, 0.7, 0.6 and 0.2 for Indonesia, Nigeria, Philippines and Pakistan, respectively. In this period, China, which had lowered its underweight rate to 17% by 1994, experienced a decline at the rate of 1% per year. The prevalence (%) of stunting among underfives in 1994 was 53, 50 and 43 in India, Pakistan and Nigeria, respectively. The rate of decline for stunting in this period was 1% for India and 0.5 and 1.3 for Nigeria and Pakistan. And China, with a stunting prevalence of 32% in 1994, saw a 2.1% decline per year in this period.
